# Heart-Type Fatty Acid Binding Protein (H-FABP): Relationship with arterial ıntima-media thickness and role as diagnostic marker for atherosclerosis in patients with ımpaired glucose metabolism

**DOI:** 10.1186/1475-2840-10-37

**Published:** 2011-05-02

**Authors:** Basak Karbek, Mustafa Özbek, Nujen Colak Bozkurt, Zeynep Ginis, Askın Güngünes, İlknur Öztürk Ünsal, Erman Cakal, Tuncay Delibası

**Affiliations:** 1 Department of Endocrinology and Metabolism, Dışkapı Yıldırım Beyazıt Teaching and Research hospital, Ankara, Turkey; 2 Department of Biochemistry, Dışkapı Yıldırım Beyazıt Teaching and Research hospital, Ankara, Turkey

## Abstract

**Background:**

Heart type fatty acid binding protein (H-FABP) has been closely associated with acute coronary syndrome, cardiac abnormalities, stroke, and obstructive sleep disorder in previous studies. The aim of this study was to evaluate and compare the serum H-FABP levels and carotid artery intima-media thickness (CIMT) between patients with prediabetes and control subjects.

**Research design and methods:**

We measured serum H-FABP levels in 58 prediabetic patients, 29 with impaired fasting glucose (IFG) and 29 with impaired glucose tolerance (IGT) and 28 age-, sex- and body mass index-matched control subjects using a sandwich enzyme-linked immunosorbent assay (ELISA), and in order to measure CIMT, all participants underwent high-resolution B-mode ultrasonography.

**Results:**

Serum H-FABP levels were significantly elevated in pre-diabetic patients when compared with that of control subjects (IFG: 32.5 ± 34.2 ng/dL, IGT: 45.4 ± 45.8 ng/dL, control: 16.8 ± 14.9 ng/dL; p = 0.011). The difference in means of H-FABP levels between patients with IGT or IFG and control subjects was significant (p = 0.010 and p = 0.009, respectively). CIMT was higher in the pre-diabetic groups compared with the control group (IFG: 0.6 ± 0.1, IGT: 0.6 ± 0.1, control: 0.5 ± 0.1; p < 0.001), and H-FABP level was positively correlated with CIMT (p < 0.001, rho = 0.626).

**Conclusion:**

Our results indicate that patients with pre-diabetes are at increased risk for cardiovascular disease. In addition, serum H-FABP levels could represent a useful marker for myocardial performance in patients with IFG and IGT.

## Background

Abnormalities of glucose metabolism are most commonly diagnosed using the threshold criteria for impaired glucose tolerance (IGT) or impaired fasting glucose (IFG) established by the American Diabetes Association (ADA) and the World Health Organization (WHO) [1-3]. These two pre-diabetic states were initially recognized as conditions associated with increased risk of progression to type 2 diabetes mellitus. In addition to the elevated glucose levels, pre-diabetes is associated with the classic cardiovascular risk factors, such as obesity, hypertension, and dyslipidemia [4-6]. IGT and IFG differ from one another in many aspects, including the associated cardiovascular risk [7]. Whereas ample evidence points to a strong relationship between IGT and cardiovascular morbidity and mortality, it is still unclear whether there is a relationship between IFG and cardiovascular disease [8,9].

Heart-type fatty acid binding protein (H-FABP) is abundant in the cytosol of cardiomyocytes, and transports fatty acids in these cells. It is a powerful regulator of the mitochondrial beta-oxidative system in the heart [10]. Molecular size and intracellular location are important factors determining the kinetic release of cardiac biomarkers. Small cytoplasmic molecules such as H-FABP (15 kDa) appear earlier (2-4 h) than larger ones, which are mainly associated with myofibrils, as cardiac troponins (6-12 h) [11]. Therefore, H-FABP has been used as a diagnostic marker for acute coronary syndrome (ACS). Other studies have demonstrated that serum levels of H-FABP are increased in patients with hypertrophic and dilated cardiomyopathy, heart failure, stroke, obstructive sleep apnea, and pulmonary embolism [12]. Carotid artery intima-media thickness (CIMT) is an intermediate phenotype for early atherosclerosis. High CIMT values have been associated with cardiovascular disease, coronary atherosclerosis, and related risk factors in cross-sectional studies [13,14]. Further, since CIMT can be measured relatively simply and noninvasively, it represents a useful diagnostic tool.

The aim of the present study was to compare serum H-FABP levels, CIMT, and cardiovascular risk factors of IFG and IGT patients with those of controls having normal glucose tolerance (NGT). In addition, to discern any possible differences between the two pre-diabetic groups, values for these three parameters in IFG patients were compared with those in IGT patients.

## Materials and methods

The present study was performed at the Endocrinology Department of Diskapi Yildirim Beyazit Training and Research Hospital, during the period January 2010-July 2010. Fifty-eight patients with pre-diabetes (29 with IGT, mean age: 46.9 ± 10.4 years, and 29 with IFG, mean age: 45.2 ± 10.3 years) along with 28 control subjects with NGT, matched for age, gender, and body mass index (BMI), were enrolled. The demographic characteristics of the case and control subjects are shown in Table 1. The study was approved by the Hospital Ethical Committee and informed consents were obtained from all participants before the study procedures.

**Table 1:** Demographic characteristics and biochemical data for patients with impaired fasting glucose (IFG), patients with impaired glucose tolerance (IGT), and control subjects

	IFG (n = 29)	IGT (n = 29)	Control (n = 28)	p
**Gender, n (%)**				
Female	23 (79.3)	23 (79.3)	23 (82.1)	0.953
Male	6 (20.7)	6 (20.7)	5 (17.9)	
**Age (Years), mean ± SD**	45.2 ± 10.3	46.9 ± 10.4	44.2 ± 8.1	0.575
**BMI (kg/m^2^), mean ± SD**	27.9 ± 2.4	28.6 ± 3.6	28.6 ± 4.1	0.652
**Waist/hip ratio, mean ± SD**	0.90 ± 0.07	0.87 ± 0.07	0.85 ± 0.08	0.047
**Hypertension, n (%)**				
Absent	28 (96.6)	27 (93.1)	28 (100.0)	0.770
Present	1 (3.4)	2 (6.9)	0 (0)	
**Smoking, n (%)**				
Absent	26 (89.7)	24 (82.8)	25 (89.3)	0.784
Present	3 (10.3)	5 (17.2)	3 (10.7)	
**Total cholesterol level (mg/dL), mean ± SD**	184.9 ± 27.2	187.6 ± 34.9	198.1 ± 23.3	0.110
**LDL level (mg/dL), mean ± SD**	117.7 ± 40.3	105.9 ± 32.6	118.2 ± 23.7	0.204
**HDL level (mg/dL), mean ± SD memean ± SD**	47.9 ± 10.2	52.2 ± 27.3	51.3 ± 11.7	0.533
**TG level (mg/dL), mean ± SD**	120.9 ± 38.9	148.0 ± 68.9	131.6 ± 48.2	0.364
**Fasting blood glucose level (mg/dL), mean ± SD**	111.1 ± 5.9	105.1 ± 8.5	92.3 ± 5.0	<0.001

**Insulin level, mean ± SD**	11.9 ± 4.8	13.1 ± 5.4	9.10 ± 4.2	**0.008**
**HOMA-IR level, mean ± SD**	3.4 ± 1.4	4.1 ± 1.7	2.1 ± 1.0	**<0.001**

HOMA-IR: Homeostatic model assessment BMI: Body mass ındex

All participants underwent a standard 75 g oral glucose tolerance test (OGTT) after 12 h fast, with venous glucose sampling at 0 min and 120 min. Categories of glucose tolerance were defined according to 2006 WHO criteria [15]. IFG is defined by an elevated fasting plasma glucose (FPG) concentration (≥100 and<126 mg/dL). IGT is defined by an elevated 2-hour plasma glucose concentration (≥140 and<200 mg/dL) after a 75-g glucose load on the oralglucose tolerance test (OGTT) in the presence of an FPG concentration <126 mg/dL. Low-density lipoprotein cholesterol (LDL-C), high-density lipoprotein cholesterol (HDL-C), total cholesterol (TC), triglycerides, and fasting serum insulin were estimated. Weight and height of the subjects were measured, and BMI and homeostasis model assessment of insulin resistance (HOMA-IR) were calculated. All participants underwent high-resolution B-mode ultrasonography. All scans and image measurements were carried out by the same investigator, who was blinded to the risk factor status of the participants. All patients were interviewed using a standard questionnaire, including demographic characteristics, concomitant disease, use of medications that could affect blood glucose and H-FABP levels and smoking history, and they underwent physical examination. Patients with a history of ACS, heart failure, pulmonary embolism, stroke, cardiomyopathy, renal disease and immunological diseases, or those who were under treatment for these diseases, were excluded from the study. The control group composed of volunteers who did not have any history of cardiological disease, ACS, heart failure, pulmonary embolism, stroke, cardiomyopathy, renal disease, immunological diseases, and diabetes mellitus.

### Heart-type fatty acid binding protein

The human H-FABP ELISA is a ready-to-use solid-phase enzyme-linked immunosorbent assay based on the sandwich principle. Samples and standards are incubated together with peroxidase-conjugated second antibody in microtiter wells coated with antibodies recognizing human H-FABP. During incubation human H-FABP is captured by the solid bound antibody. The secondary antibodies will bind to the captured human H-FABP. The peroxidase conjugated antibody will react with the substrate, tetramethylbenzidine (TMB). The enzyme reaction is stopped by the addition of oxalic acid. The absorbance at 450 nm is measured with a spectrophotometer.

### Statistical analyses

The Statistical Package for Social Sciences (SPSS) version 15.0 for Windows was used for the statistical analyses. Frequency tables for categorical variables and descriptive statistics (mean, standard deviation, median, minimum, maximum) for the numerical variables were generated. Providing the crosstab statistics for the inter-group categorical comparisons, significance levels were examined using a chi-square test. A t-test was performed to determine differences among groups for the normally distributed data. For non-normally distributed data, the Mann-Whitney U test was performed for two-group comparisons and the Kruskal-Wallis test was used for multi-group comparisons. In order to measure dependence between groups that were not normally distributed, Spearman's rho coefficient was calculated. A p value < 0.05 was considered statistically significant.

## Results

**Figure 1:**
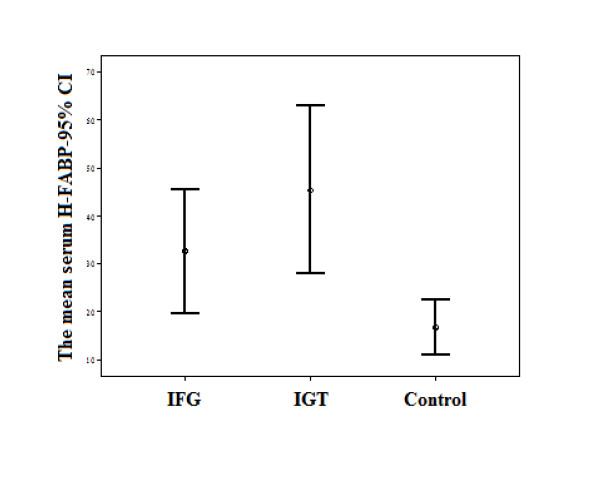
**Mean serum heart-type fatty acid binding protein (H-FABP) levels in patients with impaired fasting glucose (IFG) and impaired glucose tolerance (IGT), and in control subjects**.

Demographic characteristics and biochemical parameters of the patients with IGT, the patients with IFG, and normal control subjects are shown in Table 1. Serum H-FABP levels were significantly elevated in pre-diabetic patients compared with control subjects (IFG: 32.5 ± 34.2 ng/dL, IGT: 45.4 ± 45.8 ng/dL, control: 16.8 ± 14.9 ng/dL; p = 0.011, Figure 1). The difference in means of H-FABP levels between patients with IGT or IFG and control subjects was significant (p = 0.010 and p = 0.009, respectively; Table 2). We compared the serum H-FABP levels of pre-diabetic groups; Although the IGT group had a higher serum H-FABP level than the IFG group, the difference was not significant (p = 0.732).

**Table 2:** Serum heart-type fatty acid binding protein (H-FABP) levels in patients with impaired fasting glucose (IFG), patients with impaired glucose tolerance (IGT), and control subjects

Study Groups	H-FABP	
	
	Mean ± SD	p
**IFG**	32.5 ± 34.2	0.011
**IGT**	45.4 ± 45.8	
**Control**	16.8 ± 14.9	

	**Difference of Means ± SEM**	**p**

**IFG-IGT**	-12.9 ± 10.7	0.732
**IFG-control**	15.8 ± 6.9	0.009
**IGT-control**	28.7 ± 9.0	0.010

The mean insulin resistance test (HOMA-IR) values of the IFG group, IGT group, and control group statistically differed from each other, the values were 3.4 ± 1.4, 4.1 ± 1.7, and 2.1 ± 1.0, respectively (p < 0.001). However, HOMA-IR values were not correlated with H-FABP levels.

**Figure 2:**
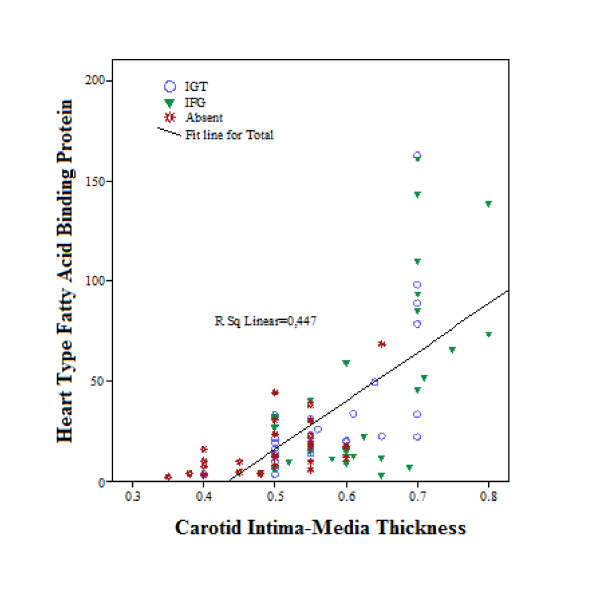
**Relationship between *carotid intima-media *thickness and heart-type fatty acid binding protein levels in patient and control groups**. IGT: impaired glucose tolerance, IFG: impaired fasting glucose, Absent: control group (no prediabetic condition detected).

CIMT was significantly higher in the pre-diabetic groups than in the control group (IFG: 0.6 ± 0.1, IGT: 0.6 ± 0.1, control: 0.5 ± 0.1; p < 0.001). However, there was no significant difference between the CIMT values of patients in the two pre-diabetic states. H-FABP was positively correlated with CIMT (Figure 2). In the 86 participants included in the study, a strong relationship was found between serum H-FABP and CIMT (p < 0.001, rho = 0.626). IFG and IGT groups were examined separately; There was a significant relationship between H-FABP and CIMT (IFG group: p < 0,001 rho:0.765 IGT group: p < 0,001 rho:0,613).

No significant correlation was identified between H-FABP levels and age, gender, BMI, or other biochemical parameters.

## Discussion

The present study confirms that serum levels of H-FABP are increased in patients with pre-diabetes, and, for the first time to our knowledge, demonstrates that elevated circulating serum levels of H-FABP may provide important prognostic information in patients with IFG and IGT. These two pre-diabetic states were initially recognized as conditions associated with increased risk of progression to type 2 diabetes mellitus. A number of studies have demonstrated that pre-diabetes is a state of subclinical inflammation, procoagulation, and endothelial dysfunction [4-6]. The two pre-diabetic states differ from one another in many aspects, including the associated cardiovascular risk. Whereas there is ample evidence pointing out a strong relationship between IGT and cardiovascular morbidity and mortality, the relationship between IFG and cardiovascular disease is still unclear and controversial [8,9]. An increased understanding of the cardiovascular risk associated with the two categories of pre-diabetes would be of great benefit for the development of screening programs and preventive strategies for subjects with IFG and IGT.

H-FABP is a cytoplasmic protein with a molecular weight of 15 kDa, which mediates the passage of fatty acids from the plasma membrane to sites of lipid synthesis. It has been reported that H-FABP is a potent inducer of cardiac myocyte hypertrophy, stimulating an increase in cell surface area, protein synthesis, and c-jun expression [16]. Furthermore, H-FABP has been reported to be a new specific serum biomarker for acute myocardial infarction [17,18]. Viswanathan et al. [19] have demonstrated that the prognostic value of elevated H-FABP is additive to that of troponin in low- and intermediate-risk patients with suspected ACS. H-FABP could therefore be of value as a marker of myocardial ischemia, even in the absence of frank necrosis. Orak et al. [20] found that for patients admitted to emergency service (ES) with chest pain, H-FABP was more sensitive and specific than troponin I and creatinine kinase myocardial band (CK-MB) in the early diagnosis of acute coronary syndrome. Kyung Su Kim et al [21] confirm that; the initial H-FABP measured by quantitative POCT has a better diagnostic value than initial myoglobin or initial CK-MB as an adjunct to the initial cardiac troponins for the early diagnosis of MI. Łukasz Figiel et al [22] also showed superior sensitivity of h-FABP compared to glycogen phosphorylase BB (GP-BB) and cardiac troponin T (cTnT) in patients with ACS. H-FABP has high diagnostic accuracy in early non-ST-segment elevation ACS.

Previous studies have shown that H-FABP is not only a marker of acute myocardial infarction, but of other conditions as well. Boscheri et al. [23] have reported that H-FABP significantly predicts mortality in patients with intermediate risk for pulmonary embolism. H-FABP could therefore also represent a novel prognostic parameter enabling the optimization of management strategy in the very difficult population of pulmonary embolism patients who are at intermediate risk. Akbal et al. [24] reported that patients with metabolic syndrome (MetS) have increased serum levels of H-FABP, indicating its promise as a marker for detection of cardiac injury during the early asymptomatic period in patients with MetS. Glatz JF et al. reported that experimental diabetes induces a marked increase of the FABP content of rat heart and suggests that this protein is involved in the enhanced fatty acid utilization by the diabetic heart [25]. In our study, serum H-FABP levels were significantly elevated in pre-diabetic patients compared with control subjects (IFG: 32.5 ± 34.2 ng/dL, IGT: 45.4 ± 45.8 ng/dL, control: 16.8 ± 14.9 ng/dL; p = 0.011). The difference in means of H-FABP levels between patients with IGT or IFG and control subjects was significant (p = 0.010 and p = 0.009, respectively). Our findings also, suggest that an elevated circulating level of H-FABP is not only a highly sensitive and specific marker of myocardial damage, but is also an important prognostic determinant in patients with pre-diabetes. Also, an elevated circulating level of H-FABP can reflect early identification of subclinical atherosclerosis in patients with pre-diabetes.

CIMT is an intermediate phenotype for early atherosclerosis. As it can be measured relatively simply and noninvasively, it is well suited for use in large-scale population studies. Ultrasonic measurements correlate well with histology [26], and an increased CIMT is associated with vascular risk factors and the presence of more advanced atherosclerosis [27-30], which includes coronary artery disease [31-33]. CIMT is being increasingly used for cardiac risk stratification and as an endpoint in intervention studies. An important precondition for the application of CIMT is that it can predict future risk of clinical vascular events. A number of longitudinal studies have examined the relationship between CIMT and future events, most frequently the incidence of cardiac events (specifically, myocardial infarction (MI), angina pectoris, and coronary intervention) and cerebrovascular events (stroke or transient ischemic attack) [26-33]. More recently, a clinical study in type-2 diabetics by Djaberi et al. [34] reported a significant relationship between CIMT and abnormal cardiac perfusion. Ito and coworkers [35], Escobedo and colleagues [36], and Poppe and associates [37] confirm that vascular imaging can be effectively used to detect subclinical disease in type 2 diabetics and potentially predict cardiovascular risk. Einarson TR and coworkers [38] who published a meta-analysis of data from 15,592 patients that were described in 11 papers, found significant relationship between postprandial glucose levels and CIMT, which have both been associated with adverse cardiovascular outcomes. In our study, CIMT was significantly higher in the pre-diabetic groups than in the control group (IFG: 0.6 ± 0.1, IGT: 0.6 ± 0.1, control: 0.5 ± 0.1; p < 0.001). However CIMT was similar in both pre-diabetic states. In contrast to previous studies, the risk appeared to be the same in the two categories of pre-diabetes. Finally, we also found a positive correlation between H-FABP levels and CIMT (p < 0.001, rho = 0.626).

In conclusion, to the best of our knowledge, the present study is the first case-control study in which significant alteration in serum H-FABP levels was detected in patients with pre-diabetes. H-FABP levels were increased in patients with IFG and IGT, and serum H-FABP levels were positively correlated with CIMT. There was no difference between patient and control groups in other known cardiovascular risk factors, including age, sex, cholesterol panels, HT, and smoking. However, serum H-FABP levels in patients were high and there was a positive correlation with IMT. H-FABP may therefore represent a marker for early atherosclerosis in pre-diabetic patients.

Further studies are required to investigate the relationship between H-FABP levels and long-term development of cardiac injury and atherosclerosis in patients with pre-diabetes.

## Competing interests

The authors declare that they have no competing interests.

## Authors' contributions

First authorship is shared between BK and MÖ as both had equal contribution to study concept and design, search of the literature and the drafting and revision of the manuscript. Other authors participated in enrolling patients in the study and discussion. All authors read and approved the final manuscript.
